# Expression of Programmed Death‐Ligand 1 in Cervical Cancer: Correlation With Histologic Subtypes and Clinicopathological Features

**DOI:** 10.1002/iid3.70464

**Published:** 2026-05-06

**Authors:** Zahra Vahedpoor, Amirhasan Matini, Habibollah Rahimi, Majid Lotfinia, Hossein Motedayyen, Leyla Sadat Hejazi

**Affiliations:** ^1^ Autoimmune Diseases Research Center Kashan University of Medical Sciences Kashan Iran; ^2^ Trauma Research Center Kashan University of Medical Sciences Kashan Iran; ^3^ Physiology Research Center Kashan University of Medical Sciences Kashan Iran; ^4^ Department of Obstetrics and Gynecology Kashan University of Medical Sciences Kashan Iran

**Keywords:** cervical cancer, immunohistochemistry, programmed death‐ligand 1 (PD‐L1), squamous cell carcinoma

## Abstract

**Background:**

Cervical cancer remains a significant global health challenge, with squamous cell carcinoma (SCC) and adenocarcinoma representing its predominant histologic types. Programmed death‐ligand 1 (PD‐L1) expression in cervical cancer has been implicated in tumor immune evasion, yet its prognostic significance remains unclear. This study aimed to evaluate PD‐L1 expression in cervical cancer and its association with clinicopathological features and patient survival.

**Methods:**

This study included formalin‐fixed, paraffin‐embedded tissue samples from forty‐seven patients with cervical cancer. PD‐L1 expression was assessed by immunohistochemistry (IHC) and correlated with clinical data, HPV status (determined by p16 IHC and HPV DNA PCR/genotyping), and 2‐year survival outcomes. Statistical analyses included Fisher's exact test and Kaplan–Meier survival analysis.

**Results:**

PD‐L1 was expressed in 61.2% of cases, predominantly in keratinizing SCC (*p* = 0.006) and tumors with diffuse HPV positivity (*p* < 0.001). PD‐L1 expression significantly correlated with advanced FIGO stage (*p* = 0.03) but not with age, vascular invasion, and 2‐year survival. No significant survival difference was observed between PD‐L1 positive and negative groups.

**Conclusions:**

PD‐L1 is frequently expressed in cervical carcinoma, especially keratinizing SCC and HPV‐diffuse tumors, but its expression was not associated with 2‐year survival. The heterogeneous expression and complex tumor‐immune interactions suggest that PD‐L1 alone is insufficient as a prognostic biomarker. Future research integrating additional immune and molecular markers is needed to improve prognostication and therapeutic stratification.

## 1 Introduction

Cervical cancer, as a global health crisis, is one of the most common malignancies among women [1]. In 2020, its prevalence was approximately 604,000 cases, and its mortality rate was 342,000 cases worldwide [2]. The most important risk factors have been proposed for this disorder, including smoking, pregnancy and childbirth, sexual behavior, infectious diseases (viral, fungal, and bacterial infections), and other factors (family history and menopause earlier than 45 years) [3].

In the early stages, cervical cancer is often asymptomatic and may be diagnosed during screening pelvic examination. During the disease progresses, symptoms may be observed as an abnormal vaginal bleeding, unusual vaginal discharge, and pelvic pain during intercourse [4]. A foul‐smelling vaginal discharge is rarely considered as a symptom [5].

Based on the WHO classification of female genital tumors [5], squamous cell carcinoma (SCC) and adenocarcinoma are the most common types of cervical cancer [1, 5]. SCC begins in the squamous epithelial cells lining the ectocervix (the outer part of the cervix). It includes about 70‐80% of all cases of cervical cancer [5]. Persistent infection by high‐risk types of human papillomavirus (HPV), especially HPV‐16, are strongly associated with this disease [6]. Adenocarcinoma develops in the glandular cells of the inner canal of the cervix that connects to the uterus, called the endocervix. This cancer accounts for approximately 10–20% of cervical cancers. It is more likely to be missed in early screenings. Consequently, its early diagnosis is more challenging, and it may follow a more aggressive clinical course than SCC. Similar to SCC, it is related to high‐risk HPV, particularly HPV‐18. Therefore, vaccination against HPV and cervical cancer screening programs are effective ways to prevent the disease [1].

Programmed death‐ligand 1 (PD‐L1) is an immune checkpoint protein that plays an important role in tumor immune evasion [7, 8]. It is expressed on tumor cells and tumor‐infiltrating immune cells such as macrophages, dendritic cells, and lymphocytes [9]. In cervical cancer, PD‐L1 expression is relatively common, with prevalence varying widely (30–70% of cases). PD‐L1 expression has been reported to correlate with disease progression and patient outcomes, although findings are inconsistent [10]. The increased expression level of PD‐L1 is largely associated with tumor stage (advanced FIGO stage), larger tumor size, nodal metastasis, and parametrial invasion. The prognostic role of PD‐L1 in cervical cancer is less clear. Previous studies have reported that high PD‐L1 expression is linked to shorter overall survival and poor prognosis, due perhaps to its role in immune suppression [10, 11, 12, 13]. On the contrary, some reports have indicated that PD‐L1 expression, especially when accompanied by high tumor‐infiltrating lymphocytes (TILs) density, associates with a longer survival [14]. Others have shown that PD‐L1 expression is not associated with clinicopathological parameters such as parity, clinical findings, disease stage, size of lesion, lymph node status, and overall survival in cervical cancer patients [13, 15].

Although many studies have indicated that PD‐L1 is frequently expressed in cervical cancer and often correlates with advanced stage, nodal metastasis, and HPV‐driven tumor biology, existing discrepancies in the literature highlight the need for further investigation to clarify its exact clinicopathological and prognostic role. Therefore, this study investigated the expression of PD‐L1 in patients with cervical cancer and its correlation with clinicopathological features and survival outcomes.

## 2 Materials and Methods

### 2.1 Study Population

A total of 47 women with cervical cancer were recruited among those referred to the Gynecology Department of Shahid Beheshti Hospital, Kashan, Iran, from September 2011 to March 2018. Cervical carcinoma was diagnosed by a specialist according to clinical and pathological criteria [16, 17]. Disease stage was determined according to the International Federation of Gynecology and Obstetrics (FIGO, stages 0–IV). Inclusion criteria included: (1) female patients with cervical carcinoma confirmed by pathology; (2) non‐pregnant women. Exclusion criteria were: (1) patients with normal cervical pathology; (2) pregnant women; and (3) patients who had received any prior treatment for cervical cancer (including chemotherapy, radiotherapy, immunotherapy, and surgery) before sample collection. This study was approved by the Ethics Committee of Kashan University of Medical Sciences (IR.KAUMS.MEDNT.REC.1399.148). The written informed consent was collected from volunteers before study initiation. The initial sample size was calculated using survival parameters from a previous study [18]. Using the Log‐rank test in STATA version 13, based on an assumed 2‐year survival difference (s1 = 0.5000, s2 = 0.7071, alpha = 0.05), a target sample size of 47 patients was estimated to achieve a power of approximately 62%. Due to archival tissue limitations (detailed in Results), the final analyzable cohort consisted of 31 patients.

### 2.2 Study Procedure

Formalin‐fixed, paraffin‐embedded (FFPE) tissue blocks from pretreatment diagnostic cervical biopsies were retrieved from the pathology archive. Sections of 4 μm thickness were prepared. Hematoxylin and eosin staining of cervical tissues was performed. The optimal slide for each case was selected based on tumor tissue volume and staining quality and was subsequently used for immunohistochemical (IHC) staining. For HPV analysis, consecutive sections were used for both immunohistochemistry (as described in the Results) and HPV genotyping (detailed below). The diagnosis, histologic type, and subtype of carcinoma were confirmed by a pathologist.

### 2.3 Immunohistochemistry Procedure

Formalin‐fixed, paraffin‐embedded (FFPE) tissue sections were stained using the Master Polymer Plus Detection System (Peroxidase) (Incl. DAB Chromogen) and Rabbit anti‐human PD‐L1 Monoclonal Antibody (Abcam, UAS, Cat number: ab237726, Clone: CAL10), following the manufacturer's protocol [19]. PD‐L1 immunoreactivity was evaluated using a semi‐quantitative scoring system based on membranous staining of tumor cells. The Tumor Proportion Score (TPS) was employed, defined as the percentage of viable tumor cells showing partial or complete membranous staining of any intensity. A case was considered PD‐L1 positive if TPS ≥ 1%. Staining intensity was also recorded as follows: 0 (negative), 1+ (weak), 2+ (moderate), and 3+ (strong). Only membranous staining was considered; cytoplasmic staining was disregarded. A case was considered PD‐L1 positive if TPS ≥ 1%. This scoring method was adapted from previously published PD‐L1 evaluation protocols in cervical and other solid tumors [20, 21] and was applied by an experienced pathologist blinded to clinical data.

### 2.4 HPV Assessment

HPV status was evaluated using a dual‐method approach for comprehensive characterization. Surrogate marker p16 IHC was performed using the CINtec® p16 Histology kit (Roche mtm laboratories, Germany) on FFPE sections following the manufacturer's instructions, with p16 overexpression (defined as strong and diffuse nuclear and cytoplasmic staining in ≥ 70% of tumor cells) considered positive for high‐risk HPV transcriptional activity. Concurrently, HPV DNA genotyping was conducted. DNA was extracted from FFPE tissue sections using the QIAamp DNA FFPE Tissue Kit (Qiagen, Germany), and its quality and concentration were assessed via NanoDrop spectrophotometry. Genotyping for high‐risk HPV types was performed via PCR amplification of the L1 region using consensus primers GP5 + /GP6 + , followed by analysis of PCR products for specific high‐risk genotypes (e.g., HPV‐16, ‐18, ‐31, ‐33, ‐45) using the INNO‐LiPA HPV Genotyping Extra line probe assay (Fujirebio, Belgium). Samples with known HPV genotype served as positive controls, and no‐template reactions as negative controls. Based on the combined results, HPV status for correlation analysis was categorized as: Negative (both p16 IHC negative and HPV DNA negative), Focal (limited/patchy p16 positivity < 70% and/or focal/low‐load HPV DNA detection), and Diffuse (strong diffuse p16 overexpression ≥ 70% coupled with positive high‐risk HPV DNA detection).

### 2.5 Statistical Analysis

The association between PD‐L1 expression and clinicopathological characteristics of the patients was assessed using Fisher's exact test. Survival analysis was performed using Kaplan–Meier curves, and comparisons between groups were conducted with the Log‐rank test. All analyses were carried out using STATA software version 13. A *p*‐value of less than 0.05 was considered statistically significant.

## 3 Results

### 3.1 Patient Descriptions and Clinicopathological Features

A total of 47 patients were initially identified. Thirteen cases were excluded due to a lack of tissue blocks, two because of insufficient tissue for further sectioning, and one due to pregnancy. Finally, 31 cases were included in the final analysis. The mean age of the patients was 51 years (range: 27–79 years). At the 2‐year follow‐up after diagnosis, 22 patients (70.9%) were alive, while 9 (29.0%) had died. Vascular invasion was observed in 18 cases (58.0%). Other clinical characteristics are summarized in Table 1.

**Table 1:** Clinical Features of Patients.

Variables	Clinical findings	
Cancer types	Adenocarcinoma: 5 (16.1%)	Subtypes
		Mucinous: 2 (6.4%)
		Villoglandular: 3 (9.6%)
	SCC: 26 (83.87%)	Subtypes
		Keratinizing: 10 (32.2%)
		In situ carcinomas: 5 (16.1%)
		Non‐keratinizing: 11 (42.3%)
FIGO staging	Stage 0 (in situ): 9 (36%)	
	Stage I: 8 (32%)	
	Stage II: 3 (12%)	
	Stage III: 1 (4%)	
	Stage IV: 2 (8%)	
Depth of vascular invasion	Zero: 5 (16.1%)	
	< 5 mm: 4 (12.9%)	
	≥ 5 mm: 22 (70.9%)	
Inflammatory response	Mild: 10 (32.2%)	
	Moderate: 13 (41.9%)	
	Severe: 8 (25.8%)	

### 3.2 PD‐L1 Expression and HPV Status

**Figure 1:**
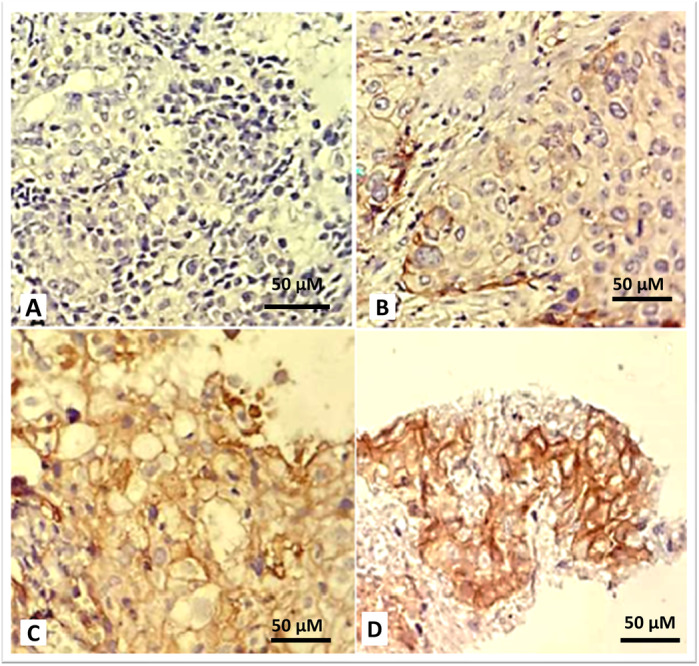
Representative IHC staining of PD‐L1 in biopsy samples (400×). Immunostaining was performed using an anti‐PD‐L1 antibody. Staining intensity was scored based on membrane staining patterns. (A) Score 0: No membranous staining. (B) Score 1 + : Faint and incomplete membranous staining. (C) Score 2 + : Moderate, complete membranous staining. (D) Score 3 + : Strong and complete membranous staining. Scale bars represent 50 µm.

PD‐L1 staining intensity was negative in 12 (38.7%), weak (1+) in 9 (29.0%), moderate (2+) in 9 (29.0%), and strong (3+) in 1 (3.3%) case (Figure 1). PD‐L1 staining was observed in ≥ 50% of tumor cells in 7 cases (22.5%), in < 50% of tumor cells in 12 cases (38.7%), and was absent in 12 cases (38.7%).

Based on the dual‐method assessment (p16 IHC and HPV DNA genotyping), HPV status was categorized as negative in 14 (45.1%), focal in 5 (16.1%), and diffuse in 12 (38.7%) cases.

### 3.3 Correlation Between PD‐L1 Expression and Clinicopathological Parameters

Overall, PD‐L1 expression was positive (TPS ≥ 1%) in 19 of 31 cases (61.2%). The mean age in PD‐L1–positive patients was 51.6 ± 14.1 years compared to 51.6 ± 13.0 years in PD‐L1–negative cases (*p* = 0.72). Seventeen (89.4%) of PD‐L1–positive tumors were SCC, and two (10.6%) were adenocarcinomas (*p* = 0.35). Within SCC subtypes, PD‐L1 expression was significantly associated with keratinizing morphology (*p* = 0.006), with 10 keratinizing (58.8%) and 6 non‐keratinizing (35.3%) tumors showing positive staining. PD‐L1 expression was also significantly correlated with FIGO stage (*p* = 0.03) and HPV staining pattern (*p* < 0.001), where all cases with diffuse HPV positivity showed PD‐L1 expression. PD‐L1 expression was not significantly associated with age, inflammatory grade, vascular invasion, invasion depth, diabetes, smoking history, and 2‐year survival.

### 3.4 PD‐L1 Staining Intensity in Invasive SCC

Among invasive SCC cases, PD‐L1 staining intensity was significantly associated with keratinization (*p* = 0.04). All tumors with strong PD‐L1 staining (2+ and 3 + ) were keratinizing SCCs. Additionally, HPV staining pattern correlated strongly with PD‐L1 intensity (*p* = 0.001); all 2+ and 3+ cases demonstrated diffuse HPV positivity. However, PD‐L1 staining intensity was not correlated to FIGO stage, inflammatory grade, vascular invasion, and invasion depth.

### 3.5 Correlation of PD‐L1 Expression Score with Clinicopathological Characteristics in Patients with Invasive SCC

Using the scoring system proposed by Garon et al. [21] and Reddy et al. [20], 5 SCCs (23.8%) were negative for PD‐L1 expression (score 0), 9 (42.9%) low positive, and 7 (33.3%) positive.

PD‐L1 expression score was significantly associated with tumor type (*p* = 0.05) and HPV status (*p* = 0.02), being higher in keratinizing SCCs and in cases with diffuse HPV staining. Other information are indicated in Table 2.

**Table 2:** Correlation of PD‐L1 Expression Score With Demographic and Clinicopathological Characteristics in Patients With Invasive SCC.

	Variables	PD‐L1 expression score			*p*‐value
		Zero (*n* = 5) *	Low positive (*n *= 9) *	Positive (*n* = 7) *	
Age	< 50 years	3 (60%)	5 (55.5%)	2 (28.5%)	0.54
	≥ 50 years	2 (40%)	4 (44.5%)	5 (71.5%)	
FIGO stage	I	1 (25%)	3 (37.5%)	5 (83.3%)	0.26
	II	1 (25%)	4 (50%)	1 (16.7%)	
	III	1 (25%)	0 (0.0%)	0 (0.0%)	
	IV	1 (25%)	1 (12.5%)	0 (0.0%)	
Vascular invasion	Yes	3 (60%)	7 (77.7%)	5 (71.42%)	0.83
	No	2 (40%)	2 (22.3%)	2 (28.57%)	
Vascular invasion depth.	< 5 mm	2 (40%)	1 (11.12%)	1 (14.8%)	0.5
	≥ 5 mm	3 (60%)	8 (88.88%)	6 (75.2%)	
Inflammation	Mild	2 (40%)	4 (44.4%)	2 (28.5%)	1
	Moderate	1 (20%)	2 (22.2%)	2 (28.5%)	
	Severe	2 (40%)	3 (33.4%)	3 (43%)	

* Zero: 0% of tumor cells are stained for PD‐L1; Low positive: 1% to around 5% of tumor cells showing PD‑L1 staining; Positive: More than 5% of tumor cells stained.

### 3.6 Clinicopathological and PD‐L1 Profiles of Fatal Cases

To provide deeper insight into the relationship between PD‐L1 expression and poor outcomes, we conducted a detailed analysis of the nine patients who died within the 2‐year follow‐up period. The clinicopathological and biomarker profiles of these cases are summarized in Table 3.

**Table 3:** Clinicopathological and PD‐L1 Characteristics of Fatal Cases (*n* = 9).

Case ID	Age	Histologic Type & Subtype	FIGO Stage	HPV IHC Status	PD‐L1 Status (TPS)	PD‐L1 Intensity	Vascular Invasion	2‐Year Status
1	68	SCC, Keratinizing	IV	Diffuse	Positive (TPS 80%)	3+ (Strong)	Yes, ≥ 5 mm	Deceased
2	55	SCC, Non‐Keratinizing	III	Focal	Negative (TPS 0%)	0	Yes, ≥ 5 mm	Deceased
3	79	Adenocarcinoma, Mucinous	II	Negative	Positive (TPS 10%)	1+ (Weak)	No	Deceased
4	62	SCC, Keratinizing	I	Diffuse	Positive (TPS 60%)	2+ (Moderate)	Yes, < 5 mm	Deceased
5	48	SCC, Non‐Keratinizing	IV	Diffuse	Positive (TPS 90%)	3+ (Strong)	Yes, ≥ 5 mm	Deceased
6	52	SCC, Keratinizing	II	Focal	Negative (TPS 0%)	0	Yes, ≥ 5 mm	Deceased
7	71	SCC, Keratinizing	I	Diffuse	Positive (TPS 40%)	2+ (Moderate)	No	Deceased
8	44	Adenocarcinoma, Villoglandular	I	Negative	Negative (TPS 0%)	0	No	Deceased
9	59	SCC, Non‐Keratinizing	III	Diffuse	Positive (TPS 5%)	1+ (Weak)	Yes, ≥ 5 mm	Deceased

Among these fatal cases, 5 out of 9 (55.6%) were PD‐L1 positive (TPS ≥ 1%), a proportion similar to the overall cohort prevalence (61.2%). However, the expression was highly heterogeneous in terms of intensity and extent. Two cases exhibited strong (3 + ), diffuse PD‐L1 expression (Cases 1 and 5, TPS 80% and 90%, respectively). Both were advanced‐stage (FIGO IV), keratinizing or non‐keratinizing SCCs with diffuse HPV positivity and deep vascular invasion, suggesting a highly aggressive, immune‐evasive phenotype.

Conversely, 4 out of 9 fatal cases (44.4%) were PD‐L1 negative (TPS 0%), indicating that tumor progression and mortality can occur independently of PD‐L1‐mediated immune evasion. These PD‐L1‐negative fatal cases included two keratinizing SCCs (one with focal HPV, one with diffuse HPV), one non‐keratinizing SCC, and one adenocarcinoma, spanning stages I to IV.

Notably, all 9 fatal cases showed at least one high‐risk feature: 7 had vascular invasion, 6 were SCC (with 4 being keratinizing subtype), and 5 indicated diffuse HPV staining. This emphasizes the multifactorial nature of poor prognosis, where factors like stage, histologic subtype, HPV status, and vascular invasion may outweigh PD‐L1 status as determinants of survival in this small cohort.

These case‐level observations highlight the non‐binary and context‐dependent role of PD‐L1 in cervical cancer outcomes. Even among patients with similar clinical outcomes (fatality), the tumor immune microenvironment—as reflected by PD‐L1 expression—can vary dramatically. This reinforces the conclusion that PD‐L1 alone is an insufficient prognostic marker and must be interpreted within a broader clinicopathological and immunologic context.

### 3.7 PD‐L1 Expression and Patient Survival

Kaplan–Meier survival analysis revealed no significant association between 2‐year survival and either PD‐L1 expression status or its staining intensity. Similarly, histologic subtype showed no significant correlation with survival outcomes.

## 4 Discussion

PD‐L1, a key immune checkpoint molecule, plays a critical role in tumor immune evasion by binding to PD‐1 on T cells and inhibiting cytotoxic immune responses [7, 8, 22]. In cervical carcinoma, especially SCC, PD‐L1 expression has been linked to tumor progression, immune escape, and poor differentiation [10]. This pathway has emerged as a promising therapeutic target, with PD‐1/PD‐L1 inhibitors showing efficacy in advanced and recurrent cervical cancer.

Our study confirms a high prevalence of PD‐L1 expression (61.2%) in cervical carcinoma, particularly within keratinizing SCC and HPV‐diffuse tumors. The central and most clinically relevant finding, however, is the lack of a significant association between PD‐L1 expression and 2‐year survival in this treatment‐naïve cohort. This dissociation between a common biomarker and outcome underscores the complexity of using PD‐L1 as an independent prognostic tool and highlights critical considerations for its predictive role in immunotherapy.

Our observed prevalence of PD‐L1 positivity (61.2%) lies within the broad range reported in previous cervical cancer studies. For instance, Grochot et al. showed variable PD‐L1 positivity and suggested that its prognostic impact may depend on the pattern of expression (focal *vs.* diffuse) and co‐expression in tumor and immune cells [23]. While our findings align with reports of frequent expression, it directly contributes to the ongoing debate regarding its prognostic value. In a larger retrospective cohort, Baek et al. similarly found no significant association between PD‐L1 positivity and overall survival in patients not treated with immunotherapy [15]. Thus, our results from a well‐characterized cohort with comprehensively assessed HPV status add weight to the growing evidence that in the absence of checkpoint blockade, tumoral PD‐L1 expression assessed by IHC may not be a robust independent prognostic factor. The inconsistency across studies may be attributed to tumor heterogeneity, variable detection methods (IHC *vs.*. RNA‐ISH), differing scoring systems, and potentially, differences in how HPV status (a key biological driver) is defined and integrated into the analysis [24].

The novel correlations we identified—specifically the strong association of PD‐L1 with keratinizing SCC and with a diffuse pattern of HPV infection—offer biologically plausible insights that may refine patient stratification. The link to keratinizing SCC may reflect a distinct, interferon‐γ‐rich tumor microenvironment that potently induces PD‐L1 expression [24]. Similarly, the strong correlation with a diffuse HPV infection phenotype supports the concept of virally‐driven immune evasion. These associations are significant because they identify patient subsets with distinct tumor biology—characterized by specific histology (keratinizing SCC) and virological profile (diffuse HPV infection)—where PD‐L1 is most prominently expressed. This could be relevant for selecting patients likely to have an active, but suppressed, immune microenvironment, a potential prerequisite for immunotherapy response.

The lack of a significant association between PD‐L1 expression and 2‐year survival in our study emphasizes the crucial distinction between its prognostic and predictive value. Our data suggest PD‐L1 is a weak prognostic marker in untreated cervical cancer. This observation is further supported by the heterogeneous profiles of our fatal cases, which underscore the multifactorial nature of poor prognosis. In our cohort, established clinicopathological determinants—such as advanced FIGO stage, aggressive histologic subtypes (e.g., keratinizing SCC), diffuse HPV infection, and the presence of vascular invasion—appeared to be stronger drivers of mortality than PD‐L1 status alone. Furthermore, these case‐level observations highlight the non‐binary and context‐dependent role of PD‐L1 in cervical cancer outcomes. Even among patients with similar clinical outcomes (fatality), the tumor immune microenvironment—as reflected by PD‐L1 expression—can vary dramatically. This supports our central conclusion that PD‐L1 alone is an insufficient prognostic marker and must be interpreted within a broader clinicopathological and virological context. Moreover, as illustrated by the detailed profiles of the nine fatal cases in our cohort, both PD‐L1‐positive and PD‐L1‐negative tumors were represented among patients with poor outcomes, reinforcing the notion that PD‐L1 expression is neither necessary nor sufficient to dictate disease aggressiveness in cervical cancer. However, its established role as a predictive biomarker for response to PD‐1/PD‐L1 inhibitors (e.g., in the KEYNOTE‐826 trial) is not diminished by our findings; rather, they clarify its clinical context [25]. PD‐L1 testing is primarily useful for therapy selection, not for predicting the natural disease course. It is important to note that even for prediction, PD‐L1 alone has limitations. Several reviews indicate it can be an unsatisfactory biomarker due to tumor heterogeneity, assay variability, and the influence of other immune modulators [25]. Therefore, our findings do not diminish the clinical utility of PD‐L1 testing but rather clarify its context: it is primarily a predictive marker for therapy selection, not a definitive prognostic marker for natural disease course. The heterogeneous expression observed in our and other studies highlights why a binary result may be insufficient; spatial distribution, CPS, and integration with other markers (e.g., CD8 + T‐cell density) are likely needed for optimal predictive power [26].

Given the limitations of PD‐L1 as an independent biomarker, there is a growing emphasis on multi‐dimensional biomarker panels for both prognostication and prediction of immunotherapy response. Other immune parameters—such as tumor mutational burden (TMB), microsatellite instability (MSI), gene expression signatures, and TILs—are being actively explored to complement PD‐L1 testing [24]. Notably, some studies report trends linking higher TMB to shorter survival, independent of PD‐L1 status, highlighting the additive value of such composite biomarkers [15]. Future research and clinical trials should therefore focus on validating integrated models that combine PD‐L1 with features of the tumor immune microenvironment and key etiological factors like HPV to improve patient stratification and outcome prediction.

Although our study has several strengths‐ including the use of pre‐treatment biopsy specimens, thereby avoiding confounding effects from therapy‐induced alterations, the focus on newly diagnosed rather than recurrent and metastatic disease, and the correlation of PD‐L1 expression with multiple clinicopathologic variables, including a rigorously defined HPV status‐ several limitations should be acknowledged. First, the small sample size limits the statistical power and generalizability of our findings. Second, the relatively short follow‐up period (2 years) may unclear potential long‐term survival associations. Third, our study employed a specific PD‐L1 scoring method (TPS) based on tumor cell membrane staining. It is important to acknowledge that different scoring systems exist—such as the Combined Positive Score (CPS), which includes immune cells, or the Tumor Area Positivity (TAP)—and the choice of scoring system can significantly influence the reported prevalence of PD‐L1 expression and its clinical correlations. This variability may limit direct comparisons with studies using alternative scoring criteria. Fourth, reliance on a single IHC method and scoring approach may not fully capture PD‐L1 heterogeneity and alternative transcript‐level regulation. Finally, the absence of data on immune infiltrates (e.g., CD8⁺ T cells, TILs) and molecular biomarkers (e.g., TMB) restricts a more comprehensive understanding of PD‐L1's prognostic significance.

Given these limitations, we recommend that future studies include larger sample sizes, extended follow‐up durations, and the application of multiplex immunohistochemistry and spatial transcriptomic techniques to better characterize the tumor–immune interface and intratumoral heterogeneity. Furthermore, concurrent evaluation of additional biomarkers—such as TMB, gene expression profiles, TILs, and interferon‐γ signatures—alongside PD‐L1 and detailed HPV characterization may provide a more accurate prediction of immune response. Assessing the predictive value of PD‐L1 (alone or in combination with these biomarkers) in patients receiving PD‐1/PD‐L1 inhibitors for cervical cancer is also warranted. Finally, the use of RNA‐ISH and transcript‐level assays may help minimize scoring variability and heterogeneity bias, as suggested by Rotman et al. [24].

## 5 Conclusion

In summary, our study provides two key contributions to the literature on cervical cancer: (1) it reinforces the high frequency of PD‐L1 expression, specifically linking it to keratinizing SCC and diffuse HPV infection, and (2) it clearly demonstrates the lack of independent prognostic value for PD‐L1 in a treatment‐naïve cohort. This highlights that while PD‐L1 is a critical predictive biomarker for immunotherapy, its expression alone does not dictate the natural prognosis of cervical cancer. Future research should focus on multiplexed biomarker panels that combine PD‐L1 with features of the tumor immune microenvironment (e.g., TILs, CD8+ cells) to improve both prognostic models and predictive algorithms for immunotherapy response.

## Author Contributions

**Zahra Vahedpoor:** methodology, writing – original draft. **Amirhasan Matini:** conceptualization, validation. **Habibollah Rahimi:** formal analysis. **Majid Lotfinia:** data curation, investigation. **Hossein Motedayyen:** validation, writing – review and editing. **Leyla Sadat Hejazi:** methodology, writing – original draft.

## Consent

Informed consent was given before taking part in the study. All authors agree to publish the article.

## Conflicts of Interest

The authors declare no conflicts of interest.

## Data Availability

The data that support the findings of this study are available from the corresponding author upon reasonable request.
